# Amyloid precursor-like protein 2 (APLP2) affects the actin cytoskeleton and increases pancreatic cancer growth and metastasis

**DOI:** 10.18632/oncotarget.2990

**Published:** 2014-12-11

**Authors:** Poomy Pandey, Satyanarayana Rachagani, Srustidhar Das, Parthasarathy Seshacharyulu, Yuri Sheinin, Naava Naslavsky, Zenggang Pan, Brittney L. Smith, Haley L. Peters, Prakash Radhakrishnan, Nicole R. McKenna, Sai Srinivas Panapakkam Giridharan, Dhanya Haridas, Sukhwinder Kaur, Michael A. Hollingsworth, Richard G. MacDonald, Jane L. Meza, Steve Caplan, Surinder K. Batra, Joyce C. Solheim

**Affiliations:** ^1^ Eppley Institute for Research in Cancer and Allied Diseases, University of Nebraska Medical Center, Omaha, NE, USA; ^2^ Department of Biochemistry and Molecular Biology, University of Nebraska Medical Center, Omaha, NE, USA; ^3^ Department of Pathology and Microbiology, University of Nebraska Medical Center, Omaha, NE, USA; ^4^ Department of Pathology, University of Colorado, Aurora, CO, USA; ^5^ Department of Biostatistics, University of Nebraska Medical Center, Omaha, NE, USA; ^6^ Current addresses: Department of Stem Cell Transplantation and Cellular Therapy, University of Texas MD Anderson Cancer Center, Houston TX, USA; ^7^ Current addresses: School of Medicine, University of Virginia, Charlottesville, VA, USA; ^8^ Current addresses: Life Sciences Institute, University of Michigan, Ann Arbor, MI, USA

**Keywords:** actin, amyloid precursor-like protein 2, metastasis, migration, pancreatic cancer

## Abstract

Amyloid precursor-like protein 2 (APLP2) is aberrantly expressed in pancreatic cancer. Here we showed that APLP2 is increased in pancreatic cancer metastases, particularly in metastatic lesions found in the diaphragm and intestine. Examination of matched human primary tumor-liver metastasis pairs showed that 38.1% of the patients had positive APLP2 expression in both the primary tumor and the corresponding liver metastasis. Stable knock-down of APLP2 expression (with inducible shRNA) in pancreatic cancer cells reduced the ability of these cells to migrate and invade. Loss of APLP2 decreased cortical actin and increased intracellular actin filaments in pancreatic cancer cells. Down-regulation of APLP2 decreased the weight and metastasis of orthotopically transplanted pancreatic tumors in nude mice.

## INTRODUCTION

Pancreatic ductal adenocarcinoma is a strikingly invasive and metastatic disease with an appallingly low survival rate [1]. Only ~20% of pancreatic cancer patients are candidates for surgical resection, and those patients who do receive resection rarely survive for long, due to metastases [2]. Standard of care therapy for metastatic pancreatic cancer patients only prolongs survival for a short time. Thus, there is a need to identify new molecular targets for treating this disease.

As shown previously by our laboratory and others, amyloid precursor-like protein 2 (APLP2) is expressed at a high level in pancreatic cancer cell lines [3-5]. According to our recent studies, APLP2 is also over-expressed in human primary pancreatic tumors, relative to its level in normal human pancreatic ductal epithelial cells [5]. These results are consistent with previous findings that APLP2 expression is increased within invasive breast cancer [6]. APLP2 expression was also demonstrated to be elevated in colorectal cancer, and APLP2 knockdown increased the susceptibility of HCT116 colon cancer cells to an apoptotic stimulus [7]. In pancreatic cancer cells, we have observed association of the major histocompatibility complex (MHC) class I molecule with APLP2 [4, 8-9]. We have found that APLP2 increases MHC class I internalization in HeLa cells, which suggests it may contribute to cancer immune evasion [4, 8-9]. Furthermore, we have shown that APLP2 assists pancreatic cancer cell survival and growth *in vitro*, and treatment of pancreatic cancer cells with beta-secretase inhibitors decreases both APLP2 cleavage and cell growth [5].

In this study, we observed elevated expression of APLP2 in human pancreatic cancer metastases. We noted that APLP2 down-regulation in pancreatic cancer cells alters the actin cytoskeleton and decreases migration and invasion. Pancreatic tumors in which APLP2 expression was down-regulated by induction of APLP2 shRNA expression were smaller and more restricted in metastatic spread. Mouse xenograft tumors having down-regulated APLP2 expression had large, abnormal, actin-containing protein complexes. Together, our findings indicate that APLP2 influences actin structures and supports specific attributes of pancreatic cancer cells (such as migration and invasion) that contribute to metastasis.

## RESULTS

### APLP2 expression is elevated in pancreatic cancer metastases

**Figure 1:**
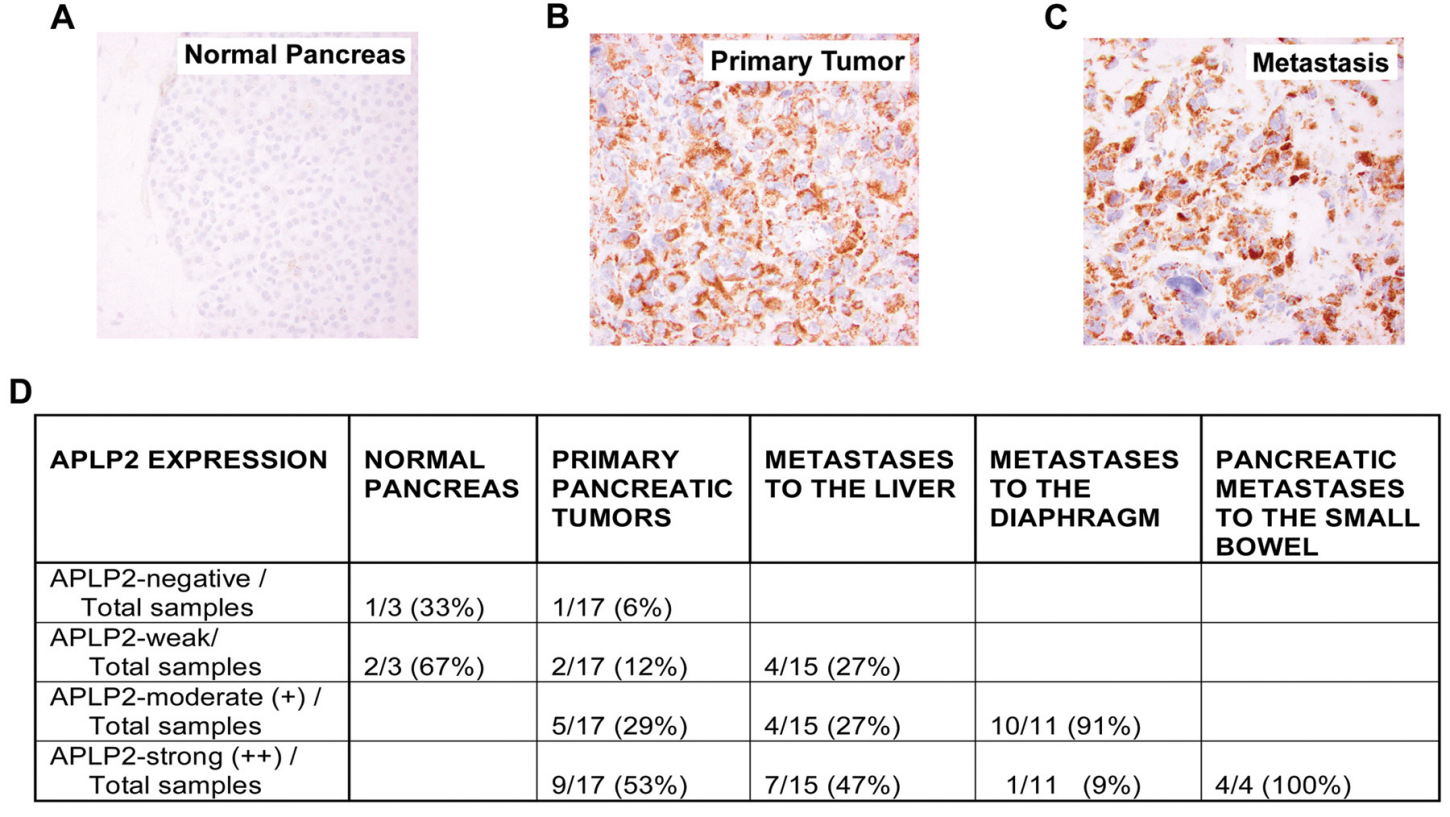
APLP2 expression is elevated in human pancreatic tumor metastases Representative immunostaining images of formalin-fixed and paraffin-embedded tissue sections were stained with anti-APLP2 antibody (EMD Biosciences, San Diego, CA) in (A) normal pancreatic tissue, (B) primary pancreatic adenocarcinoma, and (C) pancreatic adenocarcinoma metastatic to the small bowel (400× magnification). The APLP2 staining is brown, and the cell nuclei are counter-stained blue by Mayer's hematoxylin.

In this study, we investigated the expression of APLP2 in metastatic lesions from the liver, diaphragm, and small intestine of pancreatic cancer patients, as well as in primary pancreatic cancer adenocarcinomas and normal pancreatic tissue. Figure 1 shows APLP2 expression in a representative sample from normal pancreas (Figure 1A), compared to primary tumor (Figure 1B) and to metastasis in the small bowel (Figure 1C). Figure 1D indicates the percentages of samples within a tumor microarray that were scored as APLP2-negative, -weak, -moderate, or strong. Within this tumor microarray, APLP2 staining in all of the 3 normal pancreas samples was negative or weak, whereas approximately half (53%) of the 17 primary pancreatic tumors were strongly positive for APLP2 and only 6% were negative. These data are consistent with our previous report that primary pancreatic tumors tend to express increased APLP2 [5]. Extending our findings, our analysis of this tissue microarray also demonstrated that the higher level of APLP2 is maintained or elevated further in pancreatic tumor metastases, particularly in the diaphragm (100% of 11 samples were either moderate or strongly positive for APLP2 expression) and small bowel (4 out of 4 were strongly positive).

**Figure 2:**
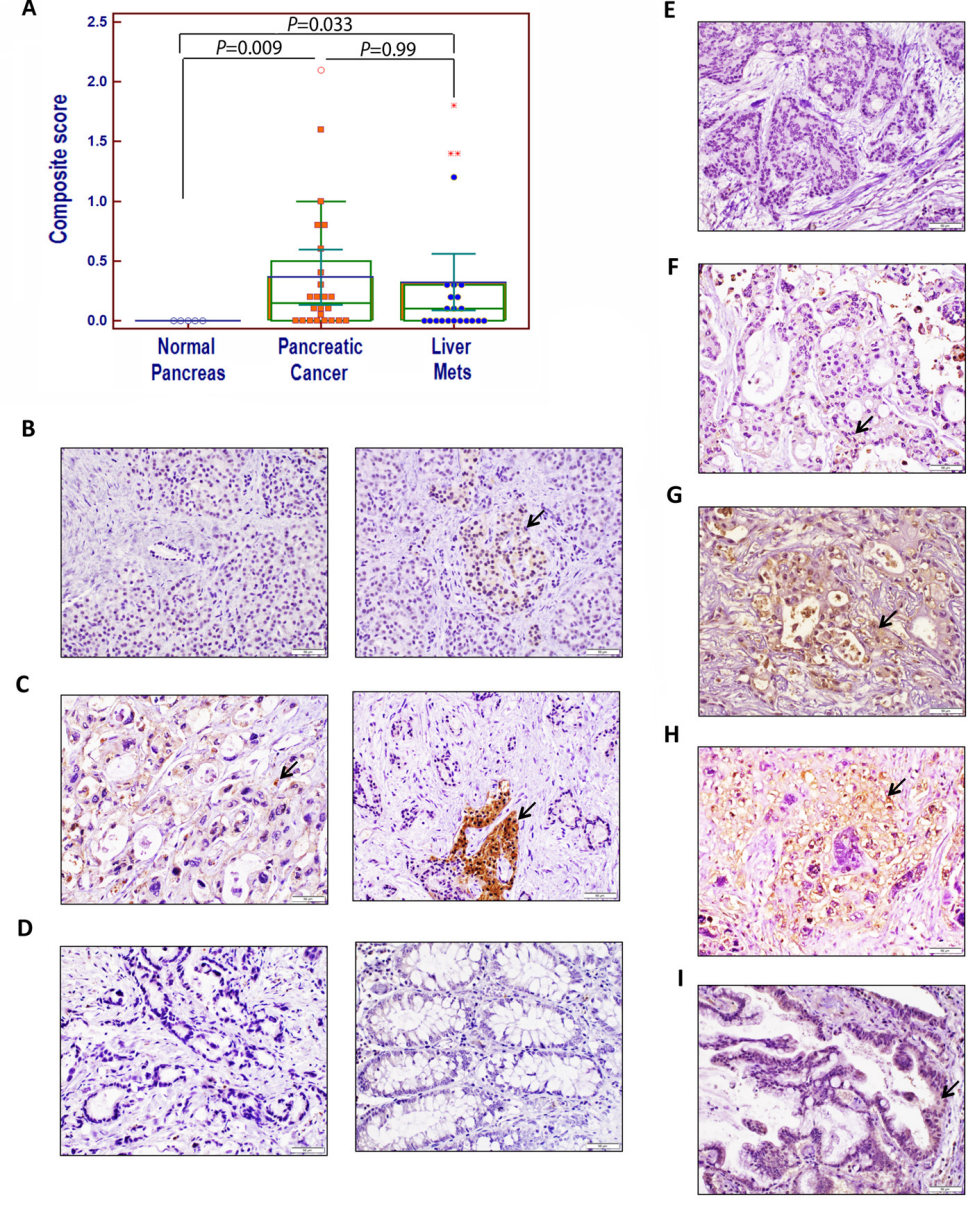
Immunohistochemical analysis of APLP2 protein expression in human pancreatic tissues, primary pancreatic cancer tissues, and patient-matched metastatic lesions in the liver and lung (A) A comparison of the composite scores for APLP2 protein expression in normal, malignant primary, and metastatic tissues from pancreatic cancer patients. Statistical analysis was done using Student's t test with the Bonferroni correction. The graphing software for the scatter plot automatically indicates out-of-range values with different symbols (open circle or asterisks). (B) The left panel presents normal pancreas with a small duct (negative for APLP2 expression), and the right panel shows normal pancreas with weak APLP2 positivity in neuroendocrine cells. (C) In the left panel, APLP2 positive expression is shown in pancreatic cancer tissue, with cytoplasmic staining in the cancer cells. The right panel shows pancreatic cancer that is APLP2 negative and islet of Langerhans that is positive (indicated by an arrow). (D) Data are displayed showing the absence of detectable APLP2 in a pancreatic cancer section (left panel) and in normal colon tissue (right panel). (E) APLP2 negative staining in a section of liver metastatic tissue is shown. (F) APLP2 weak staining in liver metastatic tissue is demonstrated. (G) APLP2 moderate staining in liver metastatic tissue is presented. (H) APLP2 intense staining in liver metastatic tissue is shown. (I) APLP2 positivity in lung metastatic tissue is displayed. The scale bar represents 50 μm. Selected areas of focal staining are indicated by the arrows.

APLP2 expression was also determined in a separate set of pancreatic cancer tissue array samples, which contained patient-matched primary and metastasis samples (Figure 2A). Among these primary pancreatic, liver and lung metastatic tumors, the frequencies of positive APLP2 expression were 66.66% (16/24), 52.17% (12/23), and 100% (1/1), respectively. We did not observe APLP2 expression in the ductal cells of any of the 5 normal pancreas samples in this set (Figure 2B). Some of the endocrine cells within normal pancreas specimens showed variable APLP2 immunoreactivity, demonstrating that APLP2 protein expression differs slightly among the cell types in the normal pancreas (Figure 2B). Figure 2C illustrates APLP2-positive (left panel) and APLP2-negative (right panel) sections of pancreatic cancer tissue (with the right panel including an islet of Langerhans that is APLP2-positive, indicated by the arrow).

It has been noted by others that macrophages have increased expression of APLP2, compared to monocytes [10]. Overall, APLP2-positive inflammatory cells were not found in significant numbers throughout the pancreatic cancer tissue sections, and all our composite scores were calculated considering only the epithelial tumor cells (and not the staining of any immune cells). In our analysis, normal colon and kidney tissue were found to lack APLP2 expression (Figure 2D shows an APLP2-negative pancreatic cancer section in the left panel, and an image of negative APLP2 staining in normal colon tissue in the right panel).

Of the 21 patients assessed for APLP2 expression in primary pancreatic tumors with matched liver metastases, 8 of 21 patients (38.1%) had positive APLP2 expression in both the primary tumor and the liver metastasis, whereas a lesser percentage (5 of 21, or 23.8%) had negative expression in both. Six of 21 (28.6%) had positive APLP2 expression in their primary pancreatic tumors but not in the corresponding liver metastases. Two of 21 patients with an APLP2-negative primary tumor specimen had positive APLP2 expression in their liver metastases. (Representative images of negative, weak, moderate, and strong APLP2 expression in liver metastases are shown in Figure 2E,F,G,H). Furthermore, we evaluated an APLP2-positive primary pancreatic cancer tissue with a matched lung metastasis (Figure 2I), as well as a matched liver metastasis, and both of the metastatic lesions were positive for APLP2 expression. No statistically significant difference was found between the APLP2 positivity in primary pancreatic cancer versus paired liver metastatic tissue (Figure 2, P=0.789).

High APLP2 expression in primary pancreatic and metastatic liver lesions was also found to be associated with lower levels of tumor differentiation: 7/8 and 1/8 strongly APLP2-positive sections were from moderately and poorly (respectively) differentiated stages of pancreatic cancer patient tissue. In contrast, of the 5 patient tissues in this series that were negative for APLP2 (in primary and liver lesions), 4/5 and 1/5 were assessed as exhibiting well differentiated and moderately differentiated stages of pancreatic cancer, respectively. Thus, these results, as well as our previous study focused on primary tumors [5], indicate association of strong APLP2 expression with poorer tumor differentiation, and therefore suggest that concordant high expression of APLP2 is likely also associated with poor prognosis.

### Pancreatic cancer cell migration is increased by APLP2

**Figure 3:**
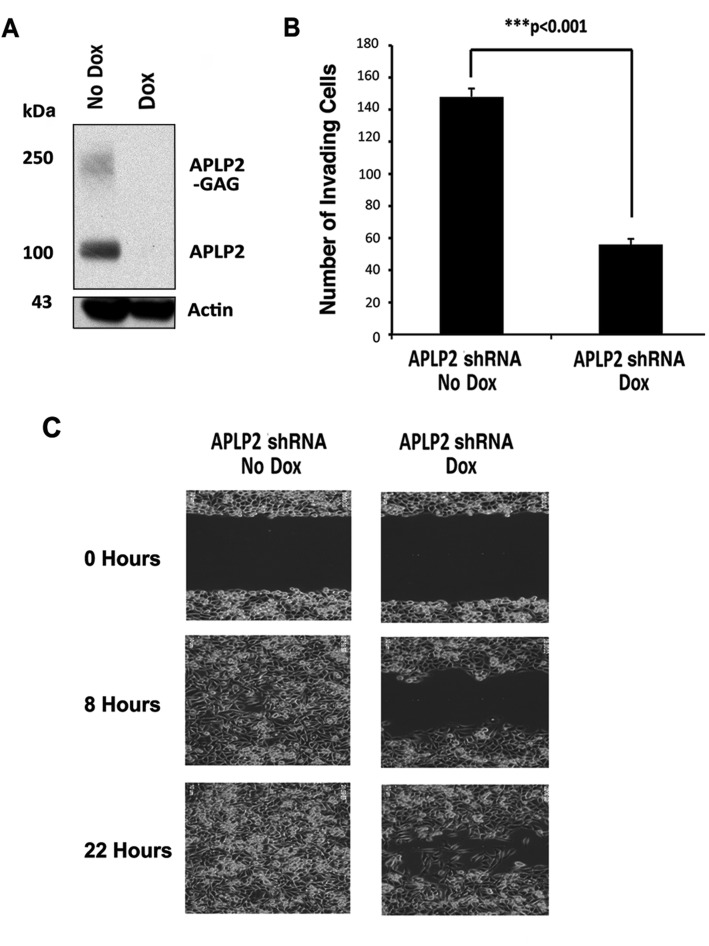
Down-regulation of APLP2 expression decreases the invasion and migration of pancreatic cancer cells (A) APLP2 down-regulation (following the addition of Dox to S2-013-APLP2-shRNA cell culture media) was verified by immunoblotting with a goat polyclonal antiserum against APLP2 (R&D Systems, Minneapolis, MN), along with an anti-actin monoclonal antibody as a loading control (Sigma, St. Louis, MO). (B) Loss of APLP2 decreases invasion. The invasion of S2-013-APLP2-shRNA cells (not cultured with Dox, or cultured with Dox) was assessed as described in the Materials and Methods. Photographs of randomly selected fields of cells that had migrated through the membranes of triplicate inserts were taken (10 for control and 10 for experimental samples), and 600-1000 total cells were counted for each type of control or experimental sample. The means and standard errors of the mean are displayed on the graph; P<0.001 for Dox versus No Dox by the Mann-Whitney test. Results shown are representative of 3 experiments with APLP2-shRNA, plus 2 with APLP2-siRNA (see Supplementary Figure 1). (C) Loss of APLP2 decreases cell migration rate. The migration rate of S2-013-APLP2-shRNA cells (not cultured with Dox, or cultured with Dox) was monitored by the approach described in the Materials and Methods. The results shown in this figure are representative of those obtained in 3 separate experiments (see Supplementary Figure 2 for videos).

To investigate the effect of APLP2 on pancreatic cancer cell mobility and invasion, we transfected the pancreatic cancer cell line S2-013 with doxycycline (Dox)-inducible APLP2-shRNA. After culture of the cells in medium containing Dox, the down-regulation of APLP2 expression was verified by immunoblotting (Figure 3A). By counting migrating cells in a chamber invasion assay, we determined that the average number of invading Dox-cultured cells expressing APLP2-shRNA (56±3.6 cells) was significantly lower than the average number of invading control cells (148±5.2 cells) (Figure 3B). Therefore, on a percentage basis (with the percentage of invading control No Dox cells set at 100%), only an average of 37.8% APLP2-knockdown cells (i.e., Dox-treated S2-013-APLP2-shRNA cells) had invaded. For confirmation, the invasion assays were also performed using transient down-regulation of APLP2 in S2-013 cells (comparing APLP2 siRNA transfection to control siRNA transfection), and the results with APLP2 siRNA were very similar to those obtained with APLP2 shRNA (Supplementary Figure 1). We also investigated the impact of APLP2 expression on the rate of S2-013 cell migration in a scratch assay, using live-cell imaging, and found that S2-013 migration was diminished by APLP2 down-regulation (Figure 3C; videos of the cell migration comparison are shown in Supplementary Figure 2).

### APLP2 regulates the organization of actin filaments in pancreatic cancer cells

**Figure 4:**
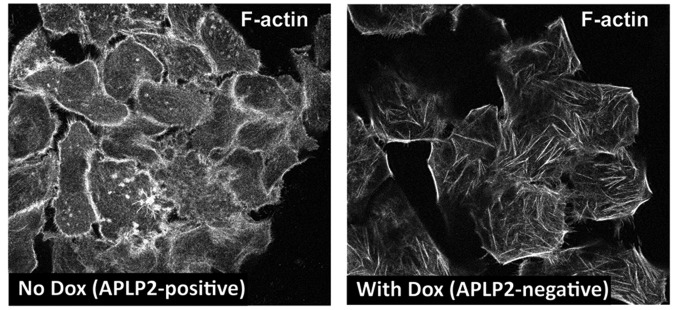
APLP2 down-regulation alters the morphology of the actin cytoskeleton S2-013-APLP2-shRNA cells (without or with APLP2 down-regulation by Dox) were grown on glass coverslips, fixed, permeabilized, and stained with rhodamine phalloidin (for F-actin filaments) (Thermo Fisher Scientific), and visualized by confocal microscopy. The images were taken with a Zeiss LSM 5 Pascal confocal microscope (Thornwood, NY, USA), using a ×63 1.4 numerical aperture lens and appropriate filters.

Actin structures are vital to the formation of invadopodia and lamellopodia, which permit tumor cell motility and metastasis [11-14]. In previous studies, APLP2 was found to associate with Fe65 adaptors that interact with the cytoskeleton through the mammalian homolog of the Drosophila Enabled gene (Mena or Enah) or the enabled/vasodilator-stimulated phosphoprotein-like protein (Evl) [15-16]. Both Mena and Evl direct actin arrangement and promote extension of actin filaments [17]. Since APLP2 influences pancreatic cancer cell invasion and migration (Figures 3B,C, Supplementary Figures 1, 2A, and 2B), we investigated whether down-regulation of APLP2 expression influenced the structure of the actin cytoskeleton in pancreatic cancer cells. We observed that Dox-treated S2-013-APLP2-shRNA cells have altered cytoskeletal morphology with substantial rearrangement of the actin cytoskeleton, as shown by staining with rhodamine phalloidin for visualization of actin filaments (Figure 4). Indeed, the APLP2-positive cells tend to display high levels of cortical actin, whereas the cells with APLP2 expression knocked down have less cortical actin and more intracellular microfilaments (Figure 4).

### APLP2 increases tumor growth in an orthotopic mouse model of pancreatic cancer

To investigate the extent to which APLP2 influences the growth and spread of tumors *in vivo*, we implanted S2-013-APLP2-shRNA-luciferase cells in the pancreas of athymic nude mice, using previously described procedures [18]. After 8 days, the mice were intraperitoneally injected with D-luciferin, imaged with the Xenogen IVIS-100, and randomized into 2 groups based on luciferase expression (such that the groups had equivalent luciferase expression). From that point on, one group of mice was given Dox in the drinking water (with 2-3% sucrose), and the other group received only 2-3% sucrose. At Day 14 and again at Day 21, the mice were again imaged with a Xenogen IVIS-100 after intraperitoneal injection of D-luciferin.

**Figure 5:**
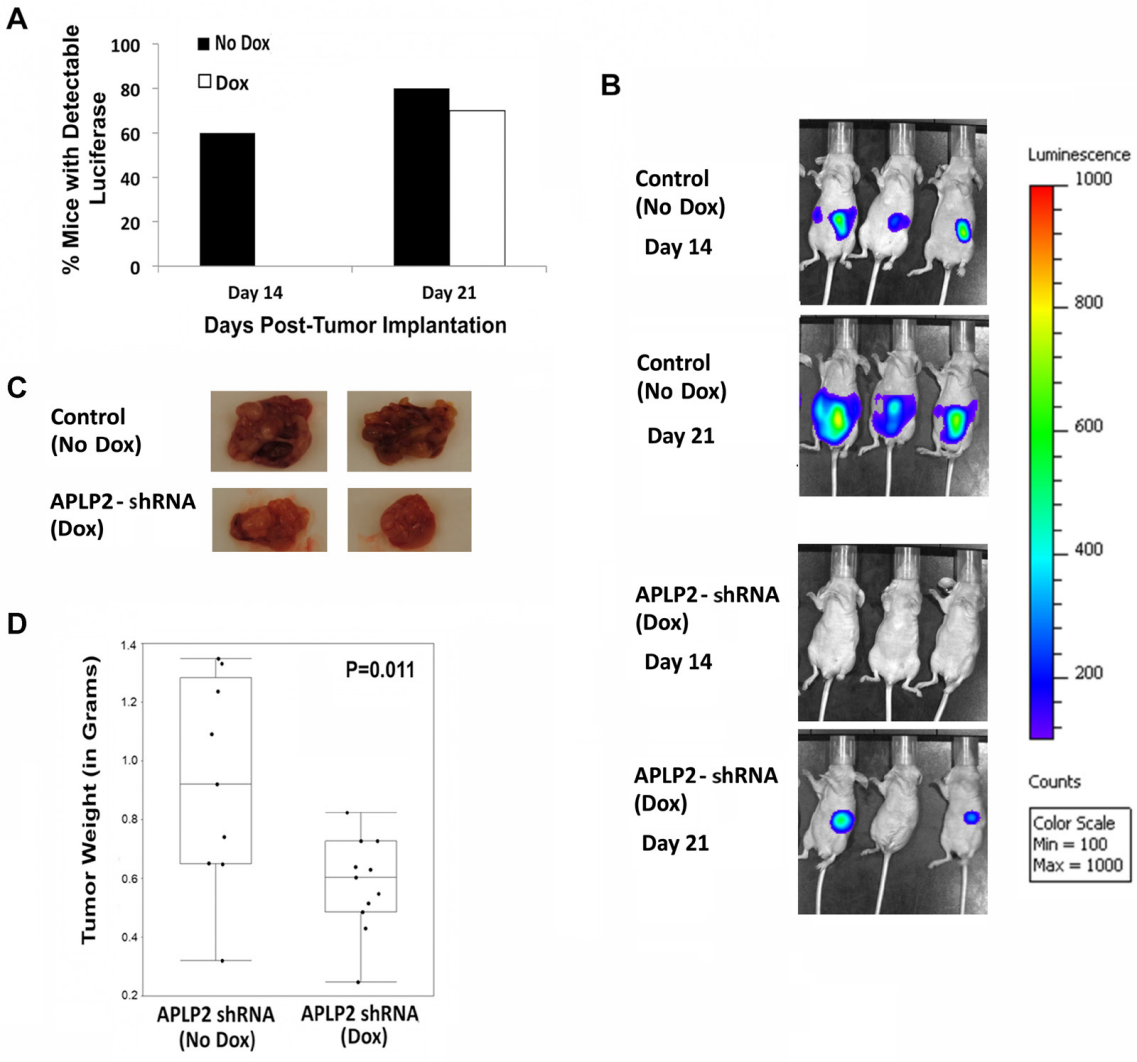
Mice implanted with S2-013-APLP2-shRNA orthotopic pancreatic tumors and then given Dox developed tumors at a slower rate and had smaller primary tumors, compared to control mice that were not given Dox S2-013-APLP2-shRNA-luciferase cells were transplanted into the pancreases of nude mice, and APLP2 shRNA expression was Dox-induced in half of the tumor-bearing mice, starting at 7 days after implantation. (A) Tumor bioluminescence was monitored on Days 14 and 21 post-tumor cell injection. At Day 14, the percentage of mice in the Dox group versus No Dox group was significantly different (P = 0.01 by Fisher's Exact Test). By Day 21, luminescence was also readily detectable in most mice in the No Dox group; there was no statistically significant difference between the Dox and No Dox groups in the percentage of mice exhibiting easily detectable luminescence at Day 21 (P = 0.99 by Fisher's Exact Test). (B) Representative images of tumor bioluminescence at Days 14 and 21 are shown. (C,D) The animals were euthanized at 30 days post-tumor cell implantation (9 mice in the control APLP2-shRNA-“off” No Dox group and 11 mice in the APLP2-shRNA-“on” Dox group), and primary tumors were resected. (C) Representative images of primary tumors resected at 30 days post-tumor cell implantation are shown. (D) The weights of all the primary pancreatic tumors resected at 30 days post-tumor cell implantation were measured. The box-and-whisker plot shows the distribution of the primary pancreatic tumor weights for each group (No Dox or Dox) around the medians. Statistical significance was assessed by the Mann-Whitney test; P=0.011.

Upon imaging, it was apparent that the knockdown of APLP2 expression significantly inhibited tumor development. Approximately 60% of the control mice had detectable tumors by Day 14 (Figure 5A). However, in the Dox-treated group, no luminescence was observed in any of the animals at Day 14 (Figure 5A). After a longer period of time (at Day 21), tumors were also perceptible in the majority of the Dox-treated mice (Figure 5A). Images of representative mice from the No Dox Group and the Dox Group at Days 14 and 21 are shown in Figure 5B.

**Figure 6:**
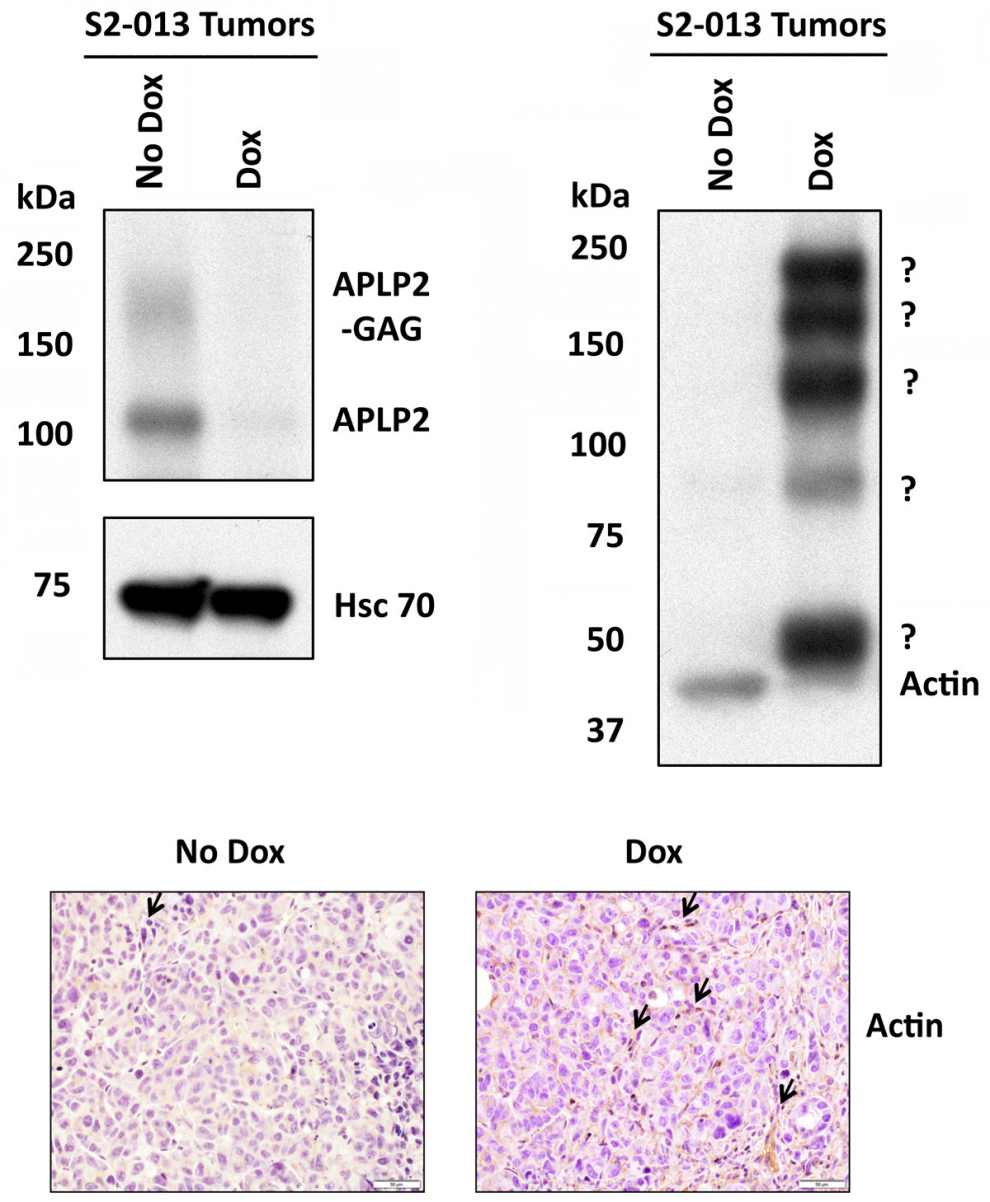
Tumors from mice implanted with S2-013-APLP2-shRNA orthotopic pancreatic tumors and then given Dox had a lower amount of actin monomers, but had an increased level of high molecular weight, covalently linked complexes containing actin APLP2 down-regulation in primary tumors of mice given Dox in the drinking water was verified by immunoblotting with a goat polyclonal antiserum against APLP2 (R&D Systems, Minneapolis, MN) (upper left immunoblot). Immunoblotting with an Hsc70 antibody (Enzo Life Sciences, Farmingdale, NY) was done as a loading control (lower left immunoblot). Blotting was performed with an anti-actin monoclonal antibody (Sigma, St. Louis, MO) to assess actin expression in the tumors (upper right panel). Immunoblots were also done on 2 other No Dox tumors and 3 additional Dox tumors, and in each case the No Dox tumors had only monomeric actin and the Dox tumors had high molecular weight structures that contained actin. Since 100% of Dox mice (n=4) demonstrated large actin complexes versus 0% for No Dox mice (n=3), by Fisher's Exact Test the difference is statistically significant (P=0.029). (Bottom panel) Immunohistochemical staining for beta-actin was performed on No Dox and Dox tumors, which revealed increased cytoplasmic staining of actin in the Dox sample (arrows indicate multiple focal areas of positive staining), compared to weak cytoplasmic actin staining in the No Dox sample. The data shown are representative images from anti-beta-actin antibody-stained sections that were obtained from 7 Dox mouse primary tumors and 1 No Dox mouse primary tumor. The scale bar represents 50 μm.

At Day 30 after tumor cell implantation, the animals were euthanized and the primary pancreatic tumors were resected and weighed. For mice that had received Dox to induce APLP2 shRNA expression, the resected primary tumors tended to be smaller (Figure 5C,D). In fact, the average weight of the Dox group tumors was approximately half of the weight for the control group (No Dox) (Figure 5D). Sections of Dox mice-derived primary tumors that were stained with APLP2 antibody showed only weak APLP2 staining, confirming *in vivo* Dox-induced knockdown of APLP2 (Supplementary Figure 3). In parallel experiments with APLP2 immunoblots of lysates from tumors obtained from 4 No Dox mice and 8 Dox mice, we again observed that APLP2 expression tended to be lower in the Dox mouse tumors (Figure 6, upper left immunoblot; data not shown), consistent with our immunohistochemistry findings.

### APLP2 is necessary for the maintenance of normal monomeric actin structure in mouse pancreatic tumors

To determine the level of actin expression in the primary tumors of the mice, we performed immunoblotting on lysates of mouse tumors. As mentioned above, immunoblotting for APLP2 verified that the APLP2 expression was reduced in the tumors of the mice that had been given Dox (Figure 6, upper left immunoblot). Hsc70 immunoblotting was also performed, as a loading control (Figure 6, lower left immunoblot). Surprisingly, immunoblotting for actin on the same tumors revealed that the APLP2 knockdown *in vivo* resulted in a decreased level of monomeric actin and the generation of high molecular weight, covalently linked complexes containing actin. Figure 6 (upper right panel) presents an actin immunoblot, with the expected single actin band at ~42 kDa in the No Dox lane. In contrast, the Dox lane shows a substantially lesser amount of actin monomers, accompanied by the appearance of large bands at approximately ~50, ~90, ~130, ~170, and ~210 kDa that are recognized by the anti-actin antibody. The exact nature of the large actin-containing protein complexes in the tumors that had APLP2 expression knocked down is presently unknown. It is notable, however, that the molecular weights of the bands in the Dox lane vary by multiples of units of ~40 kDa, which suggests the possibility that the large complexes contain a protein of ~50 kDa covalently joined to 1, 2, 3, or 4 units of actin. Including the large actin-positive forms, the actin immunoblot also indicates an overall increase of actin-positive expression in the Dox tumors, which we corroborated with immunohistochemical analysis. As shown in the bottom panels of Figure 6, we observed increased cytoplasmic staining for actin in 90% of the Dox tumor cells, whereas there was only weak to negative immunoreactivity for actin in the No Dox xenograft tumor sections.

### APLP2 increases the extent of metastasis in an orthotopic mouse model of pancreatic cancer

**Figure 7:**
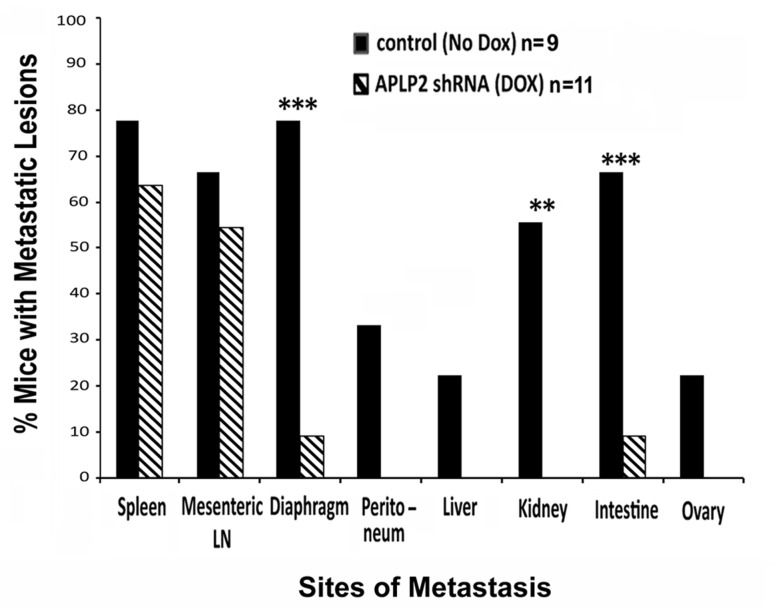
Mice implanted with S2-013-APLP2-shRNA orthotopic pancreatic tumors and then given Dox had less extensive metastases After euthanasia and dissection of the mice described in the legend for Figure 5, the percentages of tumor-bearing mice in the No Dox group or the Dox group that had metastases to various anatomic sites were recorded. The incidences of pancreatic tumor metastases were calculated as % incidence = number of mice with metastases in a particular site divided by total number of mice. Statistical significance was assessed by Fisher's Exact Test (Dox versus No Dox P-value for spleen 0.642, mesenteric lymph nodes 0.670, diaphragm 0.005, peritoneum 0.074, liver 0.189, kidney 0.008, intestine 0.017, and ovary 0.189).

We also assessed the presence or absence of metastases in various anatomic sites within both groups of mice, and found that knockdown of APLP2 in the xenografted cancer cells caused major changes in the spread of the tumors. The percentages of mice with gross metastatic lesions in the diaphragm, intestine, and kidney were dramatically lower in the mice that had received Dox (Figure 7). In addition, the group of mice that received Dox to induce the APLP2 shRNA had a trend toward having significantly lower percentages with metastases involving the spleen, mesenteric lymph nodes, peritoneum, liver, and ovary, though the differences from No Dox controls at these additional sites were not significant at P<0.05 (Figure 7).

## DISCUSSION

The findings in our current research demonstrating high APLP2 expression in pancreatic cancer metastatic lesions from patients (Figure 1 and Table 1) suggest that APLP2 may facilitate the ability of these cancer cells to metastasize. Our patient-matched APLP2 expression analysis in primary versus liver metastasis lesions predicts a possible association of APLP2 expression in both of these sites, which might contribute to poor clinical outcome. Furthermore, this study, as well as our previous one [5], highlights the involvement of APLP2 expression and its correlation with disease aggressiveness, since increased APLP2 expression correlates with a moderately or poorly differentiated stage of pancreatic cancer. Based on these findings, it may be concluded that APLP2 expression in primary pancreatic cancer and corresponding metastasis could be a factor in pancreatic cancer aggressiveness, though APLP2 expression analysis in a large cohort will be required to correlate its clinical significance with tumor stage, response to conventional chemotherapy and radiation therapy, and patient survival.

One mechanistic factor contributing to metastasis is tumor cell propensity to migrate. A previous study by another laboratory revealed that there was increased expression of APLP2 in epithelial cells that migrated in response to wounding of rat corneal epithelium [19]. Accordingly, we found that the high APLP2 expression in a pancreatic cancer cell line clearly increases its mobility and invasion capabilities (Figures 3B,C, Supplementary Figures 1, 2A, and 2B). Knockdown of APLP2 expression in tumors *in vivo* resulted in a significant slowing of tumor growth (Figure 5). Consistent with these *in vivo* findings, in our earlier analysis of APLP2′s effects on pancreatic cancer cell lines, we had also found that once APLP2 expression is down-regulated, pancreatic cancer cells cannot resume their normal growth rate [5]. Our pancreatic tumor xenograft experiments also indicated that APLP2 has a profound impact on tumor spread, significantly increasing metastasis to sites in the intestine, kidney, and diaphragm (Figure 7). These results in mice are quite consistent with our findings in human pancreatic cancer rapid autopsy samples, which demonstrated particularly high expression of APLP2 in metastases localized to the diaphragm and intestine (Figure 1).

In this study, we discovered that the down-regulation of APLP2 in pancreatic cancer cells caused major changes in the actin cytoskeleton *in vitro* (Figure 4) and in the composition of covalently linked actin-positive complexes in pancreatic tumors *in vivo* (Figure 6, upper right panel). In contrast to our noting the loss of monomeric actin in tumors, we have never observed any substantial decrease in monomeric actin in our actin immunoblots of S2-013 cells cultured *in vitro* in Dox, even when the cells were cultured in Dox for months. That such a transition from monomeric actin to high molecular weight actin-positive complexes occurs during growth of tumors *in vivo* indicates a very important function for APLP2 in maintaining normal actin structure and function in the tumor microenvironment.

Changes in actin structures are necessary for tumor cell mobilization [11-14]. Actin also has a crucial role in tumor cell division, and thus it regulates cancer cell proliferation. The C-termini of full-length and cleaved APLP2 interacts with Fe65 family proteins [15]. Fe65 proteins serve as adaptors, and the WW domain of Fe65 associates with the cytoskeleton through Mena (which is also known as Enah) or through Evl [15-16]. The expression of Mena has been noted in pancreatic cancer cell lines and tissue samples [20]. Therefore, our future investigations will explore whether the Fe65 and Mena proteins are involved in the mechanisms by which APLP2 affects the cytoskeleton. Our finding that APLP2 knockdown has a very disruptive influence on actin structure within pancreatic tumors *in vivo* (as shown in Figure 6) is particularly significant, since it has two implications for the development of novel therapies for pancreatic cancer. The first implication is that APLP2 itself may be a potential therapeutic target for pancreatic adenocarcinoma. The second implication is that APLP2 is involved in a pathway regulating actin morphology (and, thereby, pancreatic tumor migration and growth) that has not previously been delineated in detail, and which therefore may include multiple novel targets (in addition to APLP2) that could have utility in the treatment of pancreatic cancer.

We have previously shown that the growth of pancreatic cancer cells, but not non-transformed pancreatic cells, is diminished by chemical inhibitors of beta-secretase, an enzyme that generates APLP2 C-terminal fragments [5]. Cumulatively, our findings that APLP2 functions contribute to migration and metastasis, together with our previous discovery of the ability of beta-secretase inhibition to lessen pancreatic cancer cell growth [5], suggest that APLP2 could be a viable target for attacking pancreatic cancer. Thus, strategies that reduce either APLP2 expression or processing may have a therapeutic impact on deadly pancreatic cancer metastases.

## MATERIALS AND METHODS

### Ethics statement

This investigation has been conducted in accordance with ethical standards and according to the Declaration of Helsinki, as well as to national and international guidelines, and it has been approved by the authors' institutional review board and institutional animal use and care committee.

### Immunohistochemistry

The human tissue microarrays were provided for this study by the University of Nebraska Medical Center Rapid Autopsy Program, via the UNMC Tissue Procurement Shared Resource. Consent was provided by all tissue donors prior to their death, under an Institutional Review Board-approved protocol. For the immunohistochemistry experiment shown in Figure 1, a standard protocol used in the UNMC Tissue Sciences facility was employed to stain the tissues with primary anti-APLP2 antibody from Calbiochem/EMD Millipore (Billerica, MA). To produce the data shown in Figure 2, immunohistochemical staining and analysis was performed as per a published protocol [21]. Briefly, the tumor microarrays (TMAs) were initially deparaffinized using xylene and rehydrated with alcohol in a series ranging from 100% to 20% for 10 minutes each. The endogenous peroxidase activity in the human tissues was blocked by immersing the TMAs in methanol containing 3% H_2_O_2_ for an hour. The tissues were further incubated with 0.01 M citrate buffer (pH 6.8) for the antigen retrieval process. The TMAs were then incubated with 2.5% normal horse serum blocking solution (ImmPRESS Polymer Detection Reagents Kit, Vector Laboratories, Burlingame, CA) for 2 hours at room temperature. Next, the sections were incubated in a humidified chamber with primary anti-APLP2 antibody from Abcam (Cambridge, MA), diluted in 1% BSA, 0.05% Tween in Tris-buffered saline pH 7.3 at 4°C for 24 hours. After washing, the slides were incubated for 30 min at room temperature with peroxidase-labeled secondary antibody (universal anti-mouse/rabbit IgG antibody). The immunostaining was detected using peroxidase substrate detection kit with 3,3′-diaminobenzidine (Vector Laboratories, Inc.) as the chromogen. The intensity of APLP2 staining was evaluated by 2 pathologists who were blinded to the clinical data. An evaluation criterion was set for the positivity observed in the specimens, which was that the samples were considered as positive for APLP2 expression if more than 5% of the cells in the sample were stained for APLP2. Intensity of staining was evaluated as follows: negative staining = 0, weak staining = 1, moderate staining = 2, and intense or strong staining = 3. Further, a composite score was calculated as the percentage of positive cells multiplied by the intensity of staining.

For immunohistochemical analysis of the tumors generated in mice from the S2-013-APLP2-shRNA cells (with or without Dox given to the mice), the standardized protocol [21] was again used. The tissue sections were processed and stained with dilutions of anti-APLP2 antibody (R&D Systems, Minneapolis, MN) or anti-actin antibody in 1% BSA, 0.05% Tween in Tris-buffered saline pH 7.3 for 24 hours at 4°C. Following further processing of the slides (as described above), they were evaluated by a pathologist.

### Cell lines

The S2-013 pancreatic cancer cell line is a subclone of the SUIT-2 cell line that was generated from a liver metastasis, and the S2-013 cell line is known to be metastatic [22-24]. For this study, the S2-013 pancreatic cancer cells were transduced with an inducible APLP2 shRNA in the TRIPZ vector (Thermo Fisher Scientific, Pittsburgh, PA) and selected with puromycin to generate the stable S2-013-APLP2-shRNA line. Dox (at 1 μg/ml) was added to the medium to induce down-regulation of APLP2 expression in the S2-013-APLP2-shRNA cells. (By preliminary titration experiments, this concentration of Dox was determined to be the minimal concentration capable of maintaining APLP2 down-regulation.) Expression of the TRIPZ APLP2-shRNA was verified by flow cytometry at the UNMC Cell Analysis Facility by the detection of red fluorescence in the cells (which is due to expression of the TurboRFP gene in the bicistronic TRIPZ vector). The down-regulation of APLP2 following the addition of Dox was confirmed by immunoblotting.

For use in mouse xenograft experiments, S2-013 cells that also expressed luciferase (S2-013-APLP2-shRNA-luciferase) were generated by transducing S2-013-APLP2-shRNA cells with GeneCopoeia Firefly Luciferase + eGFP Lentifect Lentiviral Particles (GeneCopoeia, Rockville, MD). The cells were sorted 3 times at the UNMC Cell Analysis Facility for dual expression of RFP (the marker for APLP2-shRNA expression) and eGFP (the marker for luciferase expression) before use in experiments.

### Invasion and migration assays

To monitor invasion, S2-013-APLP2-shRNA cells (that had either been cultured with Dox, or not cultured with Dox) were seeded in RPMI medium containing 1% fetal bovine serum in the upper chambers of 24-well inserts. RPMI medium with 10% fetal bovine serum was added to the lower chambers. After 24 h of incubation (at 37ºC in 5% CO_2_), the cells in the lower chambers were stained with Diff-Quick stain (Thermo Fisher Scientific). Randomly chosen fields of cells that had invaded through the membrane were photographed.

To monitor the rate of cell migration, scratches were made with a 200-μl pipet tip in confluent cultures of cells in 6-well plates. The plates were incubated in a live-cell imaging incubator (at 37ºC in 5% CO_2_) for 24 h. The cells were visualized at 30-min intervals with an Olympus IX81 motorized inverted microscope (Olympus America Inc., Center Valley, PA), controlled via an IX2-UCB U-HSTR2 motorized system having a focus drift compensatory device (IX1-ZDC). Images were taken with a Hamamatsu ORCA ER2 CCD camera and analyzed with Slidebook version 5.0 (Intelligent Imaging Innovations, Denver, CO).

### Immunoblotting

For immunoblotting, 1×10^7^ cells were suspended in 20 mM Tris (pH 7.5), 150 mM NaCl, 5 mM EDTA, 0.5% Triton X-100, and the suspension was iced for 1 h with vortexing at intervals, and then frozen at −80°C. (For immunoblots on tumor tissue lysates, a similar process was used, beginning from matched amounts of Dox mouse tumor tissue and No Dox mouse tumor tissue that were ground with a mortar and pestle). The subsequent day, the lysates were held on ice to thaw, and microcentrifuged at 4°C for 30 min. The supernatants were kept at −80°C, then portions were mixed with 5X SDS dye consisting of 250 mM Tris-HCl (pH 6.8), 10% SDS, 30% glycerol, 0.02% bromophenol blue with 5% freshly added beta-mercaptoethanol for loading. Aliquots were boiled for 5 min and then loaded on 4→20% Tris-glycine pre-cast gels (BioRad, Hercules, CA). The samples were electrophoresed under reducing conditions for 2 h and 15 min at 90 V at room temperature, and then the proteins were transferred onto Millipore Immobilon-P membranes for 2 h at 40 V. After blocking for 2 hours at room temperature in 5% w/v non-fat dry milk in 0.05% Tween 20/phosphate-buffered saline (PBS), the blots were incubated with primary antibodies (in 5% milk solution) at 4°C overnight. All of the blots were washed at room temperature 4 times (10 min/wash) in 0.05% Tween 20/PBS, and incubated for 1 h with secondary antibodies (at a 1:10,000 dilution) in 0.05% Tween 20/PBS at room temperature. Following 3 washes (10 min/wash) at room temperature in 0.3% Tween 20/PBS, the blots were immersed in Pierce ECL Western Blotting Substrate (Thermo Scientific) and exposed to Kodak BioMax MR film (Carestream Health, Rochester, NY).

### Mouse orthotopic tumor experiments

Mouse tumor xenograft experiments were performed under a protocol approved by the Institutional Animal Care and Use Committee. The S2-013-APLP2-shRNA-luciferase cells were surgically implanted into the pancreas of 6-week-old anesthetized female athymic nude mice by the following procedure. Prior to surgery, the mice were anesthetized by i.p. injection of 350 μl of a 4:1 mixture of ketamine (from a 100 mg/ml solution) and xylazine (from a 20 mg/ml solution) diluted 10X in sterile water. The surgical site on each mouse was cleansed 3 times with Betadine Scrub^®^-soaked gauze squares and then with 70% ethanol-soaked gauze squares, beginning at the center and working out to the perimeter. Each surgical site was then sprayed with Betadine solution^®^ and outlined by a sterile drape. A 1-cm incision was made (using sterile surgical scissors) at the mid-abdomen region below the sternum in each mouse, without causing injury to the internal organs. With blunt forceps, the duodenum was pulled out slowly so that cells could be injected into the head of pancreas without causing injury and torsion. The S2-013-APLP2-shRNA cells (5×10^5^ cells in 50 μl phosphate-buffered saline) were then injected carefully into the head of the pancreas. The abdomen was closed via a 2-layer suture with 5-0 chromic catgut and soft staples. The skin staples/sutures were not removed for at least 10-14 days following the surgery.

At the 8th day post-tumor cell implantation, the mice were randomized into two groups having approximately equal tumor luminescence, as assessed by Xenogen IVIS-100 imaging at ~15 min after intraperitoneal injection of the mice with 150 mg D-luciferin/kg body weight (VivoGlo Luciferin – *In Vivo* Grade, Promega, Madison, WI). Beginning at that day, the expression of the APLP2 shRNA was induced in one of the randomized groups of tumor-bearing mice by giving the mice Dox. The Dox was delivered in the daily drinking water, with sucrose added to make the Dox solution more palatable (2 mg/ml Dox in 2-3% sucrose). The control (No Dox) mice received vehicle only (2-3% sucrose).

## SUPPLEMENTARY MATERIAL, FIGURES, TABLES




