# Supplementary material for: Amyloid precursor-like protein 2 (APLP2) affects the actin cytoskeleton and increases pancreatic cancer growth and metastasis
Source: Oncotarget. 2014 Dec 11;6(4):2064–75. doi: 10.18632/oncotarget.2990 (PMC4385836; doi:10.18632/oncotarget.2990)
Supplement: Supplementary file 1 [file oncotarget-06-2064-s001.pdf]

Amyloid precursor-like protein 2 (APLP2) affects the actin cytoskeleton and increases pancreatic cancer growth and metastasis

Supplementary Material

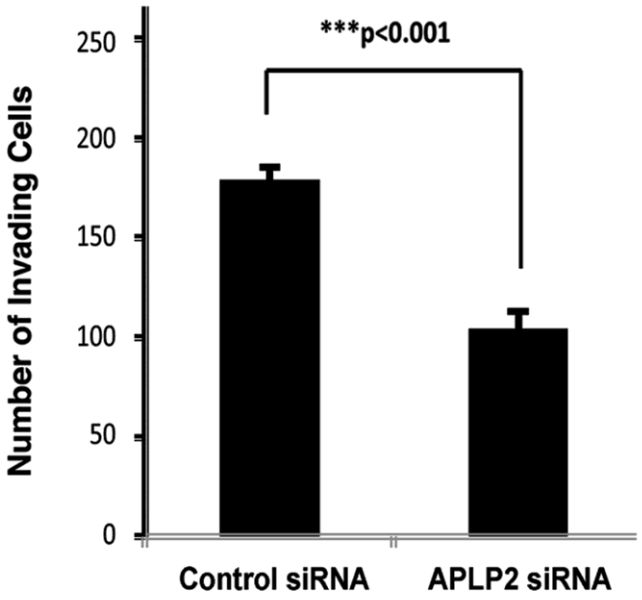

Supplementary Figure 1: APLP2 deficiency decreases invasion, as demonstrated by APLP2 siRNA transfection. These results were obtained with transient DharmaFECT 1 transfections of S2-013 cells with Dharmacon pooled APLP2-specific siRNAs, and non-targeting pooled siRNAs were used as the control (Thermo Fisher Scientific). At 48 h post-transfection, the cells were seeded in RPMI medium containing 1% fetal bovine serum in the upper chambers of 24-well inserts, and RPMI medium with 10% fetal bovine serum was put into the lower chambers. Following incubation for 24 h (at 37ºC in 5% CO_2), the cells that had migrated into the lower chambers were stained with Diff-Quick stain (Thermo Fisher Scientific). Randomly chosen fields of cells that had invaded through the membrane were photographed (5 for control and 5 for experimental samples), and ~500-900 total cells were counted for each type of control or experimental sample. The means and standard errors of the mean are displayed on the graph; P<0.001 for Dox versus No Dox by the Mann-Whitney test.

Supplementary Figure 2: Loss of APLP2 decreases the rate of pancreatic cancer cell migration. With a 200-µl pipet tip, scratches were made in confluent 6-well plate cultures of S2-013-APLP2-shRNA cells that had been grown in medium (**A**) with Dox or (**B**) without Dox. The plates were incubated for 24 h at 37ºC in 5% CO_2 in a live-cell imaging incubator. At 30-min intervals, the cells were visualized with an Olympus IX81 motorized inverted microscope (Olympus America Inc., Center Valley, PA), operated by an IX2-UCB U-HSTR2 motorized system with a focus drift compensatory device (IX1-ZDC). A Hamamatsu ORCA ER2 CCD camera was used to obtain photographs of the cells, which were analyzed with imaging software Slidebook version 5.0 (Intelligent Imaging Innovations, Denver, CO). Similar results were obtained in 3 separate experiments in which APLP2-shRNA-transfected S2-013 cells were used.

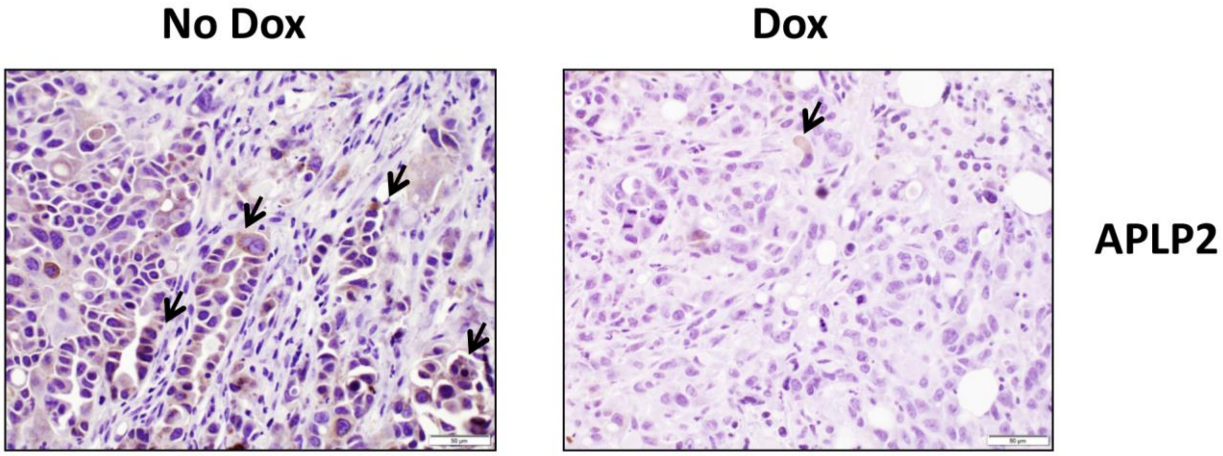

Supplementary Figure 3: S2-013-APLP2-shRNA tumor-bearing mice that received Dox had only weak APLP2 expression in the tumors. These immunohistochemistry images display the levels of APLP2 immunoreactivity in xenograft tumor sections, with weak, diffuse APLP2 staining in the Dox section, and moderate, diffuse, cytoplasmic APLP2 staining in the No Dox tumor. These data are representative of many microscopic fields observed on anti-APLP2 antibody-stained sections that were derived from 7 Dox mouse primary tumors and 1 No Dox mouse primary tumor. The scale bar represents 50 μm.
